# Human-Centered Design and Development in Digital Health: Approaches, Challenges, and Emerging Trends

**DOI:** 10.7759/cureus.85897

**Published:** 2025-06-13

**Authors:** Katerina D Tzimourta

**Affiliations:** 1 Electrical and Computer Engineering, University of Western Macedonia, Kozani, GRC

**Keywords:** brain-computer interfaces, clinical decision support systems, digital health, human-centered design, mhealth, socio-technical systems, user experience

## Abstract

Human-centered design (HCD) has emerged as a critical approach for developing digital health technologies that are usable, acceptable, and effective within complex healthcare environments. Rooted in systems theory, ergonomics, and information systems research, HCD prioritizes the needs, capabilities, and limitations of diverse user groups - including patients, clinicians, and caregivers - throughout the design and implementation process. This review synthesizes current applications of HCD in four key domains: brain-computer interfaces (BCIs), augmented and virtual reality (AR/VR), artificial intelligence (AI)-based clinical decision support systems AI-CDSS, and mobile health (mHealth) technologies. It explores design frameworks, usability strategies, and models of human-technology collaboration that contribute to successful adoption and sustained use. Ethical and legal considerations - such as data privacy, informed consent, and algorithmic fairness - are also addressed, particularly in contexts involving biometric and neurophysiological data. While HCD practices have shown substantial potential to improve digital health outcomes, their implementation remains uneven across technologies and settings. Emerging trends, including adaptive personalization, explainable AI, and participatory co-design, are identified as promising directions for the development of more inclusive, trustworthy, and sustainable digital health innovations.

## Introduction and background

Digital health technologies, including mobile applications, wearables, artificial intelligence (AI)-based systems, and neurotechnologies, are increasingly reshaping how healthcare is delivered, experienced, and managed. As these technologies become more embedded in clinical workflows and patient lives, their design must address not only technical performance but also how humans interact with, trust, and adopt them. This growing complexity has intensified the need for human-centered design (HCD), a multidisciplinary approach that ensures digital solutions align with human needs, preferences, and real-world contexts [1].

HCD is a design philosophy and process that places human needs, capabilities, and limitations at the core of system development. It emphasizes iterative design, stakeholder engagement, and contextual adaptation throughout the lifecycle of a technology. HCD extends beyond usability and visual aesthetics to encompass deeper socio-technical integration. It draws from diverse fields including systems theory [2], ergonomics [3], human-computer interaction (HCI) [4], and information systems (IS) research [5], providing a comprehensive methodology for involving users throughout the design and implementation lifecycle. In healthcare, this translates to designing for clinicians, patients, caregivers, administrators, and sometimes even regulators, each with distinct roles, constraints, and expectations [6].

Recent literature highlights that successful digital health innovations are not merely technically sophisticated, but rather contextually adapted, ethically sound, and user-accepted [7,8]. However, the adoption of HCD principles remains inconsistent across the field, with many technologies still developed in isolation from end-users or without iterative feedback mechanisms [9,10]. At the same time, new opportunities are emerging to better integrate HCD practices, especially with the rise of participatory methods, real-time usability analytics, and AI-driven personalization tools [11,12]. Despite increasing attention to HCD in healthcare, there is limited synthesis of how these principles are being operationalized in emerging digital health domains, particularly in relation to socio-technical and ethical complexity. This lack of a cross-domain perspective hinders the ability to identify shared design challenges and opportunities for more coherent implementation.

**Figure 1:**
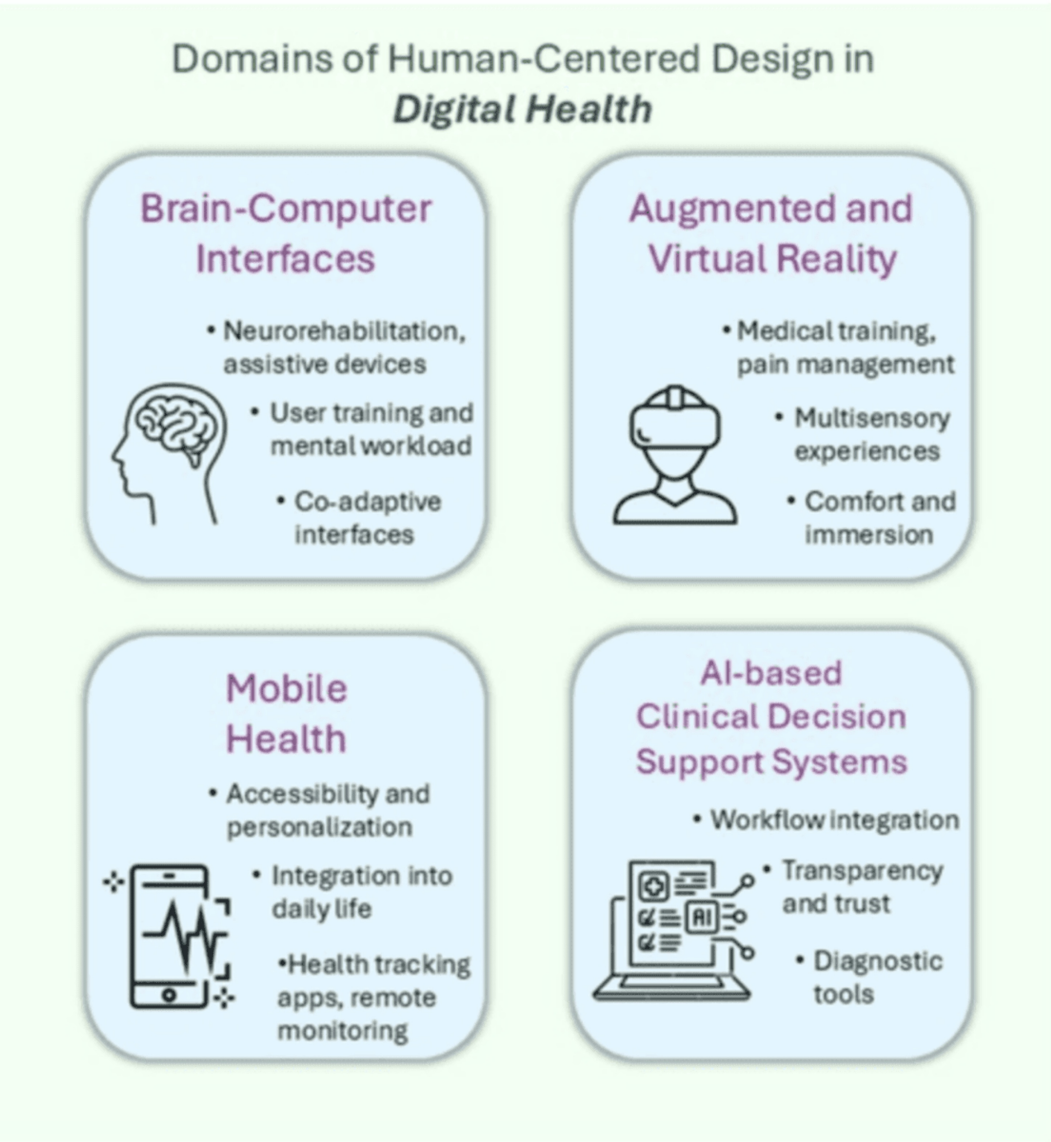
Figure summarizing four key application areas where HCD principles are implemented in digital health technologies The four areas are BCIs, AR/VR, mHealth, and AI-CDSS. For each domain, specific design priorities, such as usability, personalization, workflow integration, user training, and trust, are identified, reflecting how HCD principles guide the development of user-adapted, ethical, and effective digital health solutions AI-CDSS: artificial intelligence-based clinical decision support systems; AR/VR: augmented/virtual reality; BCI: brain-computer interface; HCD: human-centered design; mHealth: mobile health Image credits: This is an original image created by the author Katerina D. Tzimourta

This review provides a structured synthesis of how HCD principles are being applied in digital health, with a focus on four rapidly evolving domains: brain-computer interfaces (BCIs), augmented/virtual reality (AR/VR), AI-based clinical decision support systems (AI-CDSS), and mobile health (mHealth) applications (Figure 1). It aims to explore how HCD is interpreted and applied across these domains and to identify patterns, barriers, and enablers of adoption that can inform future research and development. It also examines ethical and legal concerns relevant to human-centered digital design, particularly concerning biometric and neurodata, aiming to map current practices, identify gaps, and highlight promising directions for future research and implementation. Importantly, this review extends beyond foundational theory by contextualizing HCD within emerging areas, such as BCI-AR integration, explainable AI in clinical workflows, and neuroethical data governance, offering a multidimensional perspective on human-centered innovation in digital health.

Theoretical foundations: systems, ergonomics, and information systems perspectives

Systems Theory and Socio-Technical Design

Healthcare settings are complex adaptive systems, and hence digital health tools must be designed with a systems thinking mindset. Early work in socio-technical systems theory emphasized jointly optimizing social and technical factors in organizations [3,4]. In health informatics, Sittig and Singh’s 8-dimensional socio-technical model of health IT exemplifies this approach [2]. Their framework spans hardware/software infrastructure, clinical content, HCI factors, people (all users and stakeholders), clinical workflow and communication processes, internal organizational environment (policies, culture), external rules/regulations, and system measurement/monitoring. The dimensions are interdependent, underscoring that a change in one (e.g., introducing a new AI tool) ripples through others (user roles, workflows, policies) [2]. Similarly, human factors engineers have developed systems models like the SEIPS (Systems Engineering Initiative for Patient Safety) framework, which centers the person within a work system and examines how technology fits into the patient’s “journey” across settings [5]. Such models highlight that designing for complex healthcare requires holistic, system-level thinking rather than a narrow focus on isolated device features.

Ergonomics and Human Factors

From an ergonomics perspective, HCD in digital health is rooted in designing for human abilities and limitations to improve performance, safety, and well-being. Human factors engineering principles emphasize that tools should be adapted to users (not vice versa) through iterative testing and refinement. For example, Carayon et al. note that as care becomes more distributed and complex, human factors methods must adapt to consider multiple perspectives and enable genuine user participation in design [5]. In practice, this can mean employing user-centered design techniques like task analysis, usability testing, and simulation in realistic clinical scenarios. Importantly, ergonomics reminds us that macro factors, such as team coordination, physical environment, and organizational culture, are just as critical as micro factors (user interface layout, cognitive load) in determining a technology’s success or failure. Indeed, many healthcare technology failures have been attributed to misalignment with real-world workflow or cognitive overload on users, rather than purely technical faults [6]. By treating the human, technological, and process elements as a single system, ergonomics-informed design can reduce error and improve adoption.

Information Systems and HCI Perspectives

The IS field contributes methodologies for understanding user acceptance and the socio-organizational contexts of technology use. Classic models like the Technology Acceptance Model and Unified Theory of User Acceptance highlight factors such as perceived usefulness, ease of use, and facilitating conditions as key to adoption [2]. However, these traditional models often focus on high-level determinants and may not capture the full spectrum of socio-technical interactions needed in healthcare [2]. Modern IS research advocates a human-centric IS design approach, merging HCI principles with organizational insights to ensure that digital tools truly match users’ workflows and information needs [1,7]. For example, a human-centric lens in personal health information management led Werner et al. to identify individual differences and use those insights to tailor technology design [8].

**Figure 2:**
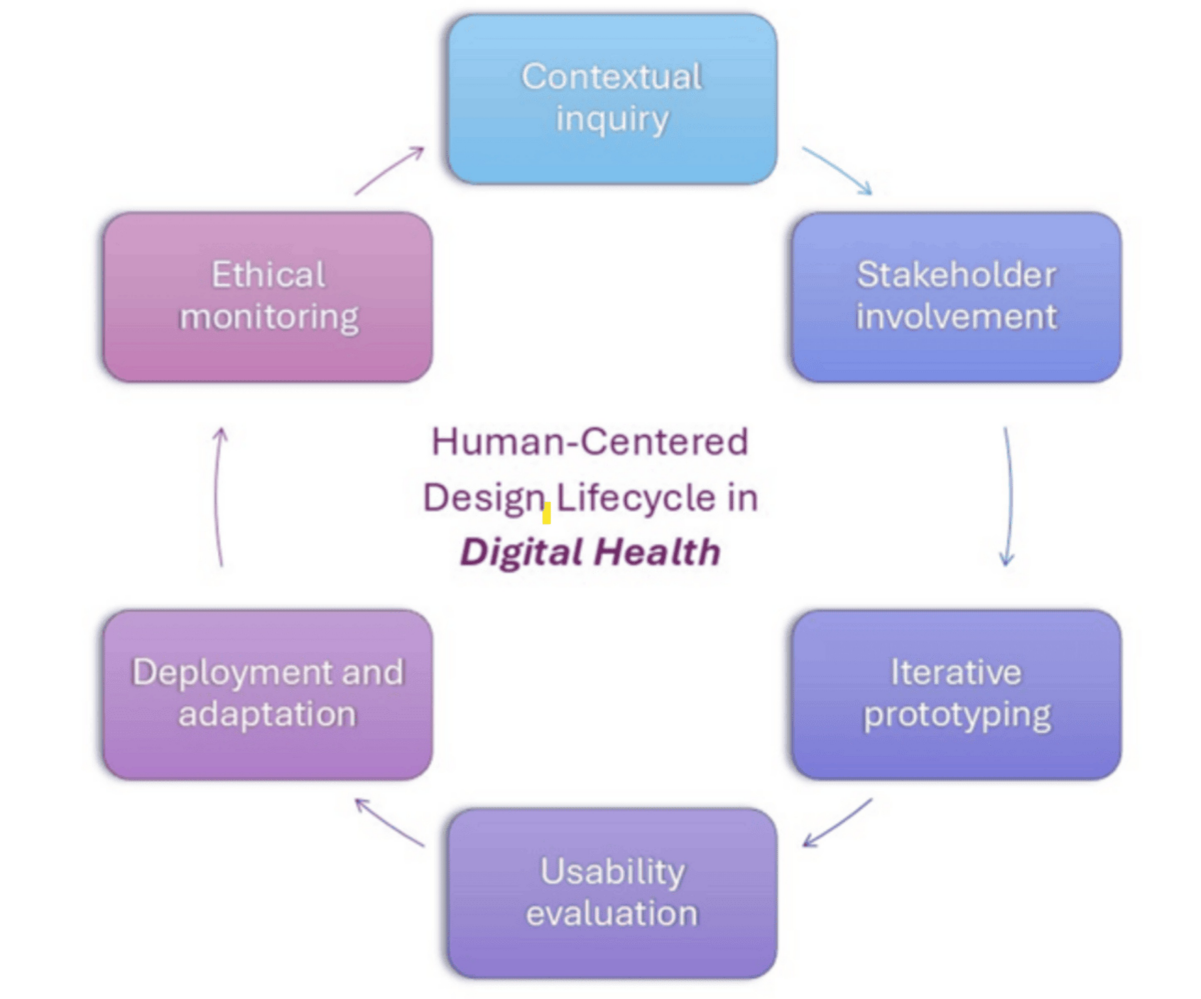
The HCD lifecycle in digital health This diagram illustrates the iterative and cyclical nature of human-centered design in the development of digital health technologies. The process typically begins with contextual inquiry and proceeds through stakeholder engagement, iterative prototyping, and usability evaluation, leading to real-world deployment. Ongoing ethical monitoring ensures the alignment of technology with user needs, safety, and societal values HCD: human-centered design Image credits: This is an original image created by the author Katerina D. Tzimourta

Participatory design and design thinking methodologies drawn from IS and HCI are increasingly applied in health innovation. These involve end-users and stakeholders early and continuously, through techniques like ethnographic fieldwork, co-design workshops, and iterative prototyping [9]. The goal is to ground the design in the realities of clinical practice and patient life, rather than making assumptions in a developer silo. In summary, theories from systems engineering, human factors, and IS converge on a common message: effective digital health solutions require human-centered, socio-technical alignment, considering everything from the cognitive ergonomics of an interface to the “big picture” of how the technology fits into a healthcare ecosystem. Figure 2 illustrates the iterative lifecycle of HCD in digital health, highlighting key phases such as contextual inquiry, stakeholder involvement, iterative prototyping, usability evaluation, deployment and adaptation, and ongoing ethical monitoring. This cyclical process reinforces the need for continuous feedback loops and ethical oversight, ensuring that digital health technologies remain aligned with user needs, clinical contexts, and societal values throughout their lifespan.

Human-centered design principles in digital health

HCD is characterized by deep empathy with users, iterative prototyping, and multi-disciplinary collaboration [9]. In the digital health context, this means engaging clinicians, patients, caregivers, and other stakeholders to ensure technology addresses real needs and pain points. Crucially, HCD in healthcare is holistic - it considers not just the end-user’s interaction with a device or app, but the surrounding sociotechnical environment [1]. As Melles et al. observe, an HCD approach takes a “holistic, systems approach towards human needs, ensuring solutions fit the dynamics of the (complex) sociotechnical system the user is part of” [10]. This often requires balancing competing demands, such as maintaining patient safety and privacy while promoting usability and accessibility.

Usability and User Experience (UX)

Usability - The ease with which people can use a technology to achieve specific goals effectively and satisfactorily - is a cornerstone of HCD. In digital health, good usability can literally be life-saving; consider an ICU nurse interacting with a patient monitor under stress. Studies show that usability and UX are key determinants of digital health success, influencing whether patients and providers adopt a tool in practice [7]. For instance, a recent review noted that despite thousands of wellness and health apps on the market, many see low sustained use, partly because they lack user-centered design and thus fail to meet user expectations or fit into daily routines [7]. HCD aims to improve metrics like efficiency, satisfaction, and error rates by tailoring design to user workflows and cognitive models. However, current practice still has gaps - a scoping review of sensor-based digital health tech found that while most studies evaluated user satisfaction and perceived ease of use, very few assessed deeper usability facets like learnability or long-term efficiency [11]. For example, 83% of devices had been evaluated for user satisfaction, but only 7% for learnability and 2% for efficiency [11]. This suggests a need to broaden UX evaluation criteria in healthcare beyond the basics, to capture how well systems support users over time and under various conditions. Furthermore, many digital health design projects focus only on the primary end-user (such as the patient) and overlook secondary users or stakeholders - one review found only ~22% of studies collected data from other users like clinicians or caregivers. HCD principles advocate involving all relevant users (patients, providers, family caregivers, etc.) to ensure the solution is usable and useful for the entire care network.

Human-Technology Collaboration

A distinctive goal in HCD for digital health is to foster effective human-technology collaboration. Rather than viewing automation or AI as replacing human roles, the emphasis is on creating synergistic partnerships between people and technology. This can mean designing interfaces that augment clinicians’ decision-making (e.g., an AI decision support that provides explainable recommendations the doctor can validate) or tools that empower patients (e.g., a self-management app that coaches a patient while respecting their personal preferences). The key to such collaboration is building trust and transparency into the system. Users are more likely to embrace a new technology if they understand its functioning and feel in control. For instance, clinicians may resist AI systems perceived as “black boxes” - studies have documented a lack of trust and adoption when an AI-driven clinical decision support is not transparent or well-aligned with clinical workflow [6]. HCD addresses this by incorporating features like explainability, user control options, and aligning technology roles with users’ mental models. It also means accounting for the social dynamics - technology should enhance communication and teamwork, not hinder it. Poorly designed systems can inadvertently create silos or divert attention away from patients, whereas well-designed ones facilitate information sharing and situational awareness. In summary, human-technology collaboration in digital health is about designing systems that leverage the respective strengths of humans and machines [6]. Achieving this requires iterative design with user feedback, ensuring that the technology becomes a seamless extension of the care process rather than a distraction or replacement.

With these principles in mind, this review examines specific application domains - BCI, AR/VR, AI-CDSS, and mHealth - to see how HCD and socio-technical strategies are being applied, what unique challenges arise, and what emerging trends are shaping each area. Although principles like usability, user involvement, and trust in AI have been widely discussed in earlier reviews, this paper focuses on how these are applied in newer and more complex areas of digital health. It explores real-world challenges and design needs in the above technologies. In addition, it brings attention to important ethical and legal questions, especially around the use of brain data, offering a more complete and up-to-date view of how HCD can support the future of digital health.

## Review

Methodology

This article presents a narrative review that aims to synthesize the current applications of HCD in selected domains of digital health. Although not a systematic review, the selection of sources followed a structured approach to ensure breadth and thematic relevance. A literature search was conducted across PubMed, Scopus and IEEE Xplore using combinations of the following keywords: "human-centered design", "digital health", "usability", "brain-computer interfaces", "augmented reality", "virtual reality", "mHealth" and "Artificial Intelligence" or "AI". The search was conducted on April 15, and articles published between 2010 and early 2025 were considered, with emphasis on peer-reviewed journal publications, review articles, and applied studies relevant to design principles and UX. 

Inclusion criteria involved relevance to HCD in at least one of the selected domains (BCI, AR/VR, AI-CDSS, mHealth), presentation of empirical or conceptual insights, and English-language availability. Excluded were studies lacking substantial reference to user-centered design or socio-technical factors (e.g., purely technical AI papers or device engineering without user evaluation), conference papers, and book chapters. The review aimed to identify cross-domain insights and recurring design challenges rather than perform an exhaustive search and a quantitative synthesis. Key references were selected iteratively to reflect both foundational works and emerging trends. Ethical, legal, and interdisciplinary issues were deliberately included to broaden the perspective on HCD implementation.

Brain-computer interfaces in digital health: a user-centric approach

BCI is an exciting and rapidly evolving category of digital health technology with the potential to revolutionize human-computer interaction for patients with severe motor impairments, as well as enable novel neurofeedback and rehabilitation therapies [12]. BCIs allow users to control external devices (computer cursors, prosthetic limbs, wheelchairs, even drones) directly via brain activity, typically measured through electroencephalography (EEG) or implanted sensors [12]. While the technical feasibility of BCI control has advanced greatly, a recurring theme in BCI research is that technical performance alone is not enough; human factors and user-centered design are crucial to make BCIs practical and acceptable in real-world healthcare settings [13]. A 2023 human factors evaluation noted that despite improvements in BCI signal accuracy and information transfer rate, the practicability of BCI is still difficult to achieve because many BCI systems do not fully consider human factors and thus fail to meet users’ expectations [13]. Key challenges include the extensive training or mental effort often required, variability in user brain signals, and the need for feedback and adaptation mechanisms as the user learns to use the BCI [12].

BCIs are characterized by a unique requirement; users must often train and be trained by the system through repeated practice and calibration. Designing a BCI thus involves a co-adaptive process, where system algorithms adapt to the user’s brain signal patterns, and the user adapts to the system’s feedback and control schema. Iterative, user-centered evaluation can quantify how users improve over time and what hurdles they face. For example, one BCI gaming study measured user adaptation by having participants play a simple EEG-controlled game in multiple sessions; users showed significant performance improvements with practice, achieving ~7.6% higher scores on average in later trials as they became more proficient [14]. The game’s design intentionally focused on user adjustment and improvement in the BCI environment, using metrics like average game score and improvement rate as outcomes [15]. Findings like these underscore the importance of providing clear feedback and an intuitive learning curve in BCI interfaces. From a design standpoint, techniques such as progressive difficulty adjustment, motivational feedback (e.g., gamification), and user-specific calibration of signal classifiers can help maintain engagement during the steep learning phase of BCI use [14]. In one case, users who played a motor-imagery based BCI game 20 times gradually achieved more reliable control, with the system detecting their brain commands with high accuracy (~98% by the end of training) [14]. Such improvements highlight that HCD for BCIs should plan for an initial adaptation period and design to support and accelerate this adaptation.

In addition to training-related factors, other human-factor issues have been identified as influential in BCI adoption. Mental and physical workload is a concern since controlling a BCI can be cognitively fatiguing, and users may become frustrated if the system is error-prone or slow to respond. A recent systematic review of BCI applications stressed that reliability and minimization of user effort are top priorities for real-world BCI deployment [12]. For instance, non-invasive EEG-based BCIs are safe and portable, but they often suffer from signal noise and require users to concentrate intensely; thus, interface design (visual cues, confirmation mechanisms, etc.) must reduce cognitive load and make the interaction as natural as possible. User satisfaction is another critical metric. Lyu et al. proposed a comprehensive HCD evaluation framework for BCIs, incorporating subjective satisfaction ratings for the BCI hardware and software, as well as mental workload assessments [13]. By systematically capturing user feedback (e.g., comfort of the EEG headset, clarity of system feedback) and workload (e.g., via NASA-TLX or similar scales), designers can identify pain points that purely technical evaluations might miss. That study provided valuable insights, suggesting that applying human factors engineering of BCI - essentially HCD tailored to BCI - can guide personalized BCI design and improve user satisfaction [13].

**Figure 3:**
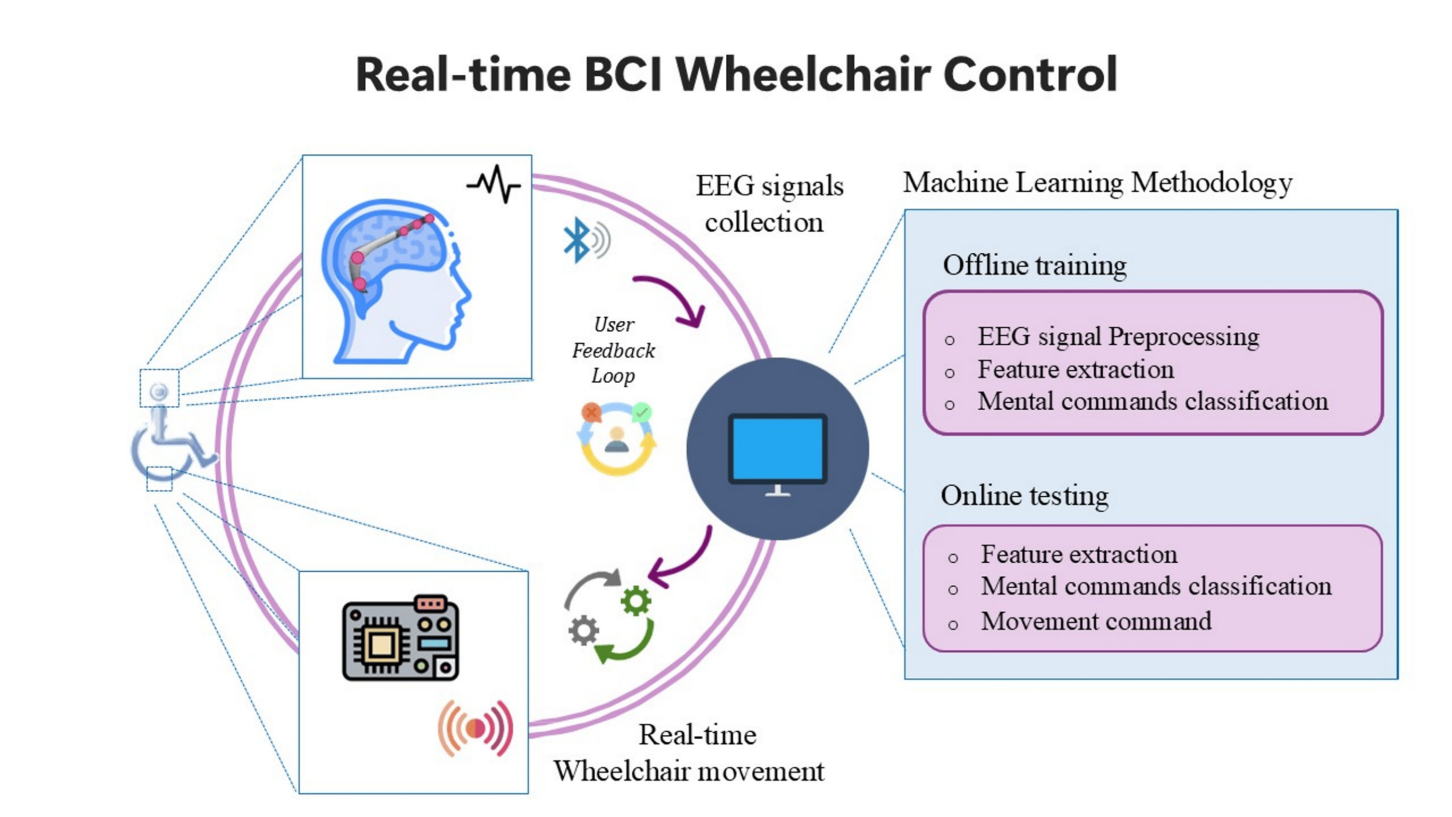
Real-time BCI control architecture with user feedback integration The diagram illustrates a typical EEG-based BCI system for wheelchair control, combining signal acquisition, machine learning processing, and real-time movement. A key feature is the inclusion of a user feedback loop, reflecting HCD principles by allowing adaptive calibration and iterative system improvement based on user interaction BCI: brain-computer interface; EEG: electroencephalography; HCD: human-centered design Image credits: This is an original image created by the author Katerina D. Tzimourta

Applications of BCIs in healthcare have been rapidly evolving, with HCD playing a pivotal role in their successful implementation. Real-world BCI applications in healthcare are expanding, and HCD is critical in each. In neurorehabilitation, BCIs combined with virtual environments have been used to help stroke patients relearn motor functions; success depends on crafting feedback that patients find meaningful and keeping them motivated during repetitive training [15]. In assistive technology, BCI-controlled wheelchairs or spellers for paralyzed users must be highly reliable and easy to operate because any complexity in the interface can render the system unusable for those with severe impairments. Figure 3 illustrates a typical real-time BCI control loop for wheelchair navigation. While technically focused, such systems must also account for human-centered aspects such as usability, user training, adaptive feedback, and ethical concerns around brain data collection and interpretation. 

A systematic review of BCI-controlled drones illustrates how new use cases bring new HCD challenges: controlling a drone via EEG in real time pushes BCI technology to be faster and more robust, and raises questions about situational awareness for the user [12]. The review of 42 studies on BCI drone control identified multiple research directions and future challenges, such as improving real-time accuracy, reducing calibration time, and ensuring user safety when controlling robots or vehicles via thought alone [12]. Another emerging trend is integrating AR interfaces with BCI control. AR can provide intuitive visual guidance to the BCI user. For example, Dillen et al. introduced a user-centric system where an AR display shows a BCI user the available commands in context, like highlighting a device to be controlled and prompting the user’s next mental action [14]. Such approaches, still in early stages, exemplify human-technology collaboration: the AR assists the human in using the BCI, effectively closing the feedback loop and potentially lowering cognitive burden. By addressing technical and human factor challenges in tandem, improving signal processing while refining user feedback and training, the next generation of BCIs aims to be not only powerful but also user-friendly and trustworthy tools in digital health.

Finally, it is worth noting that BCIs introduce novel ethical and privacy considerations. Users are understandably concerned about the privacy and security of their EEG signals and the potential misuse of brain data. Researchers have highlighted that issues of data protection and informed consent are paramount as BCI technology moves from lab to clinic [12]. Thus, a truly human-centered approach to BCI must extend beyond usability to also encompass transparent data practices and safeguards, ensuring that users feel safe and respected when plugging their brains into digital systems.

Augmented and virtual reality: human factors in immersive health technologies

AR and VR technologies are increasingly used in medicine, from surgical visualization tools and clinician training simulators with AR to VR exposure therapy for phobias and immersive rehabilitation exercises for patients. These technologies create rich interactive environments, but their efficacy depends greatly on human factors and UX design. An AR surgical headset, for example, must overlay information in a surgeon’s field of view without causing distraction or fatigue; a therapeutic VR simulation must be engaging yet not induce motion sickness or overwhelm the patient. HCD is essential to harness AR/VR benefits while mitigating their risks. As one review noted, digital health studies involving VR often evaluate aspects like user immersion, spatial awareness, and interaction with the virtual environment as proxies for UX [12]. High immersion can improve effectiveness (e.g., better pain distraction in a VR therapy), but it also raises concerns of user comfort and cognitive load. Thus, the level of immersion and complexity must be calibrated to the user’s abilities and therapeutic goals [12]. In training contexts, AR/VR designers face the challenge of balancing realism with simplicity since too much realism can introduce noise or stress, while too little can limit real-world transfer of skills.

A compelling example of HCD in VR comes from the field of neurorehabilitation. Cucinella et al. (2025) applied a human-centered participatory design process to create personalized immersive VR environments for patients with acquired brain injury [16]. Their approach involved multiple stages that included observing current rehab practices, interviewing eleven clinical experts, surveying two dozen therapists, and finally running co-design workshops with neurorehabilitation professionals. Through these steps, they gathered insights on how therapists adjust real-world exercises for patients and what features an ideal VR system should have. The outcome was the identification of key design requirements and themes for VR rehab environments; they must be Specific to the patient’s needs, Meaningful and relevant to real-life tasks, Versatile to adapt to different recovery stages, Educational, Safe, and Supportive. Notably, clinicians emphasized making the VR scenarios realistic yet customizable so that patients are neither bored nor overwhelmed. These findings illustrate the value of engaging domain experts and end-users: the resulting VR design was grounded in actual therapy workflows and patient capabilities, rather than being a tech-driven fantasy. By following HCD principles, the developers could incorporate features like adjustable difficulty, to keep patients in an optimal zone of challenge, safety measures, limiting visual stimuli for easily confused patients, and meaningful context with exercises that mimic daily activities the patient cares about. Early testing indicated that such co-created VR environments may increase patient motivation and skill transfer to real life [16]. This case study shows HCD at work, starting from the users’ world and building the technology around it, rather than vice versa.

On the other hand, immersive technologies bring unique usability challenges that designers must address. Comfort and ergonomics are paramount - wearing a VR headset or AR glasses for extended periods can cause physical discomfort (heat, weight on nose/neck) and visual strain. Ensuring devices are lightweight, adjustable, and usable for people with glasses or other assistive needs is part of HCD. Moreover, the “cybersickness” phenomenon, meaning VR-induced motion sickness or disorientation, can severely limit usability for some users. Designers use techniques like limiting sudden movements in virtual scenes, providing stationary reference frames, or matching visual and vestibular cues to reduce cybersickness. While such issues are technical at root, solving them requires an HCD mindset; iterative user testing to identify what aspects of the experience cause discomfort and creative design adjustments to alleviate them. User interface design in AR/VR also departs from conventional 2D screens. In AR, information should be contextually displayed without clutter. A common guideline is to avoid overloading the user’s visual field and to use spatial audio or minimalistic indicators to guide attention. In VR, interface elements might be embedded in the virtual world or invoked by simple gestures to maintain immersion. A narrative review of HCD in health tech found that methods like contextual inquiry and user journey mapping are especially useful for AR/VR projects, as they help designers understand the user’s physical and cognitive context at each moment [9]. For example, mapping a surgeon’s journey through a procedure can reveal when and where AR overlays would be most helpful (e.g., during a specific step when they need both hands on the patient, so perhaps voice or gaze controls are optimal for the AR system at that point).

Increasingly, digital health solutions are combining modalities such as VR with biofeedback, or AR with voice assistants to enhance UX. A systematic review by Gramouseni et al. examined EEG-based cognitive assessment in VR/AR environments, essentially merging BCI with extended reality (XR) technologies [12]. They reported promising results in measuring cognitive load and attention through EEG during VR tasks, but also pointed out practical challenges like the cumbersome setup of EEG equipment and limited sample sizes in many studies [17,18]. From an HCD perspective, this suggests that as we design multi-modal systems, we must streamline the UX; for instance, developing more user-friendly EEG headsets or integrating sensors invisibly to avoid detracting from the main AR/VR experience. The review also highlighted the need to include diverse participant groups and real-world scenarios to validate such systems [12]. This echoes a broader HCD point, and that is to test with real users in real contexts whenever possible. An AR system for paramedics, for example, should be tested in simulated emergency settings with paramedic users to uncover context-specific usability issues like gloves affecting gesture recognition or bright sunlight washing out a display.

AI-based decision support systems: trust, usability, and workflow integration

AI-CDSS are a prime example of socio-technical innovation in digital health. These systems leverage machine learning and big data to provide recommendations, predictions, or alerts aimed at improving patient care - for instance, an AI CDSS might flag early signs of sepsis, recommend personalized treatment based on prior outcomes, or assist in radiology image interpretation. The potential of AI-CDSS to enhance clinical decision-making is enormous, but so are the socio-technical challenges of implementing these tools in real healthcare settings [6]. Unlike consumer tech, where a nifty AI feature might be adopted enthusiastically, in healthcare, the bar for adoption is high: clinicians must trust the system’s output, it must fit into their busy workflow, and it must demonstrably benefit patient outcomes without introducing new safety risks. HCD and evaluation are, therefore, crucial to the successful deployment of AI-based decision support.

Clinicians’ trust in AI-CDSS has increasingly been recognized as a critical determinant of adoption and effective use. If an AI system cannot explain its reasoning or seems to contradict clinical intuition, providers are likely to ignore or override it. As one review succinctly put it, many AI-CDSS function as a black box, and end-users may resist accepting or using AI-CDSS due to a lack of trust and understanding of the system’s capability [6]. HCD approaches respond to this by incorporating explainable AI (XAI) techniques - for example, showing key factors that influenced a recommendation, or offering confidence levels and allowing drill-down into data. Co-designing AI interfaces with clinicians can reveal what kind of explanations are most meaningful to them. Furthermore, involving clinicians early in the development process not only yields interface improvements but can also increase trust as users gain insight into how the AI works and contribute their domain knowledge to its design [19,20]. Human-centered evaluation of AI-CDSS often looks at user satisfaction, perceived usefulness, trust, and workload implications [6]. For instance, Wang et al. (2023) systematically reviewed AI-CDSS studies and found that the better documented systems tended to report positive effects on efficiency and decision accuracy but also faced issues of user trust and workflow integration. They identified six major implementation challenges: a) technical limitations (e.g., insufficient accuracy), b) workflow misalignment, c) attitudinal barriers (resistance to change), d) informational barriers (data availability/quality issues), e) usability problems, and f) environmental/contextual barriers [6]. All six are essentially socio-technical issues beyond algorithms. Addressing these requires a blend of technical fixes and human-centered strategies; improving the AI model, yes, but equally improving the interface design, providing training and education, adapting workflows, and shaping policies that support AI use.

Effective integration of AI-CDSS into clinical workflows has been identified as essential for their adoption and sustained use. A well-designed AI-CDSS should feel like a helpful colleague to clinicians, not an intrusive second-guesser. This means integrating into existing clinical workflows as smoothly as possible. If a system requires extra logins, complex navigation, or disrupts the clinician’s thought process with poorly timed alerts, it will be quickly sidelined. HCD emphasizes contextual inquiry; studying how clinicians perform tasks and make decisions in order to insert the AI support at the right time and in the right manner. For example, if physicians rely on a certain workflow in the electronic health record (EHR), the AI recommendations should appear within that EHR interface and at the moment of decision, rather than in a separate application that forces context-switching. Usability testing with clinicians can catch mismatches early: Are the alerts too frequent, leading to alert fatigue? Is the language of recommendations clear or full of AI jargon? Does it take more than a few seconds to get the needed information from the system? These are the kinds of issues that a human factors evaluation would surface. Notably, workflow misalignment was cited as a significant barrier in multiple studies, meaning some AI tools, while effective on paper, did not mesh with how care was delivered day-to-day [6]. An illustrative case was an AI system for hospital fall risk prediction that provided accurate risk scores, but nurses found it unhelpful because it didn’t integrate with their existing fall prevention protocols and required them to navigate to a separate screen, a classic example where the tool wasn’t embedded in the socio-technical workflow. To fix such issues, designers might adopt user journey mapping, like charting the clinician’s end-to-end process and identifying optimal intervention points for AI prompts, or even redesign aspects of the workflow in tandem with the technology. Implementation science and HCD are increasingly intersecting here: researchers are calling for leveraging principles from both fields to ensure digital interventions are not only efficacious in trials but also actually used and sustained in practice [21].

UX has likewise emerged as a pivotal determinant of AI-CDSS success. Clinicians, especially younger ones, have rising expectations for software tools. They compare the clunky interfaces of some legacy health IT systems with the seamless UX of consumer apps. If an AI-CDSS has a confusing UI, long response times, or is not mobile-accessible, it will struggle to gain traction. HCD would focus on creating intuitive visualizations, like highlighting the patient data most relevant to the AI’s recommendation, or using color-coding for risk levels that align with medical conventions, and ensuring efficiency with minimizing clicks and allowing quick overrides or confirmations. In one study, adding a simple explanation interface to an AI sepsis alert significantly improved physician acceptance of the alerts, because it reduced the “why is it alerting?” confusion and blended into the decision process. Another dimension of UX is cognitive workload. AI systems should reduce clinician cognitive load by handling data synthesis, not adding to it. However, if not carefully designed, they can paradoxically increase the workload. For example, if clinicians feel they must double-check the AI or document reasons for not following AI advice. A human-centered evaluation by Wang et al. found that many AI-CDSS studies did measure user outcomes like workload and satisfaction and some reported improvements in efficiency like faster decisions [6]. But they also emphasized the paucity of studies focusing on UX in depth; only 20 articles in over a decade of AI-CDSS research met their inclusion criteria for analyzing human-centered aspects [6]. This indicates that more work is needed in the research community to systematically evaluate and report UX findings, not just algorithm performance.

The integration of AI into clinical settings has also redefined the nature of human-technology collaboration. Rather than replacing clinicians, the vision is often to create a human-AI team that outperforms either alone. Realizing this requires clarity on roles: what does the AI do vs. what the human does. For example, an AI might triage radiology images, but the radiologist makes the final call on difficult cases - if designed well, the AI could handle routine normal exams and free up the human expert for ambiguous ones. HCD can facilitate this by ensuring that the AI’s output is delivered with appropriate confidence indicators and options, enabling the human to calibrate their reliance on the AI. There is also a cultural and training component: clinicians need to learn how to “read” AI outputs and incorporate them into their reasoning. Some hospitals have started providing training sessions or explanation dashboards to help users get familiar with their AI tools. The concept of augmented intelligence is often used to stress that AI should augment human decision-making, not replace it, but this augmentation only works if the tool is designed to complement human cognitive processes. Encouragingly, there are emerging design paradigms like active learning or human-in-the-loop AI, where the system actively solicits user feedback on certain recommendations and improves over time, effectively learning from the human while the human learns from it. This symbiotic approach is very much in line with HCD, treating the system and user as cooperative agents in a shared workflow.

Mobile health and wearable technologies: usability and user engagement

mHealth apps and wearable devices represent the most direct interface between digital technology and health consumers. From smartphone apps for chronic disease management to wearable fitness and vital sign trackers, these technologies hold great promise for empowering patients and promoting preventive care. Yet the landscape of mHealth is notorious for its high drop-off rates and variable quality, often traceable to design issues that neglect the end-user’s perspective [22]. HCD approaches are critical to make mHealth tools that people want to use continuously and that truly improve health behaviors or outcomes.

Challenges in mHealth adoption have been widely documented, with low sustained usage and limited clinical endorsement remaining persistent obstacles. Despite the availability of hundreds of thousands of health apps, only a small proportion are regularly used by patients or recommended by healthcare professionals. One reason is that many apps are developed with a focus on medical functionality or data collection, but insufficient attention is paid to usability, engagement, and context of use. Studies have found that patients will abandon apps that are too complex, not personalized, or that do not integrate well into their daily life routines. A 2023 integrative review noted that due to high resource costs and low user adoption of mHealth apps, the cost-benefit relationship remains controversial for many digital health interventions [23]. Crucially, the authors argue that applying HCD can lead to more usable, acceptable, and effective mHealth apps, improving this cost-benefit tradeoff [7]. For example, involving target users in the design of an app can reveal seemingly simple but important requirements that, if implemented, can make the difference between an app that is engaging versus one that is burdensome. The review by An et al. goes further to recommend taking a sociotechnical lens in mHealth development, meaning developers should consider not just the user-device interaction, but also factors like the user’s environment, social support network, and the reliability/privacy of the app’s infrastructure [7]. By doing so, the sustainability of mHealth solutions can be improved, avoiding pitfalls like apps that work in a study but falter in the wild due to poor Internet connectivity or lack of integration with healthcare providers [7].

Ensuring usability and accessibility has been recognized as essential in the development of mHealth applications, particularly given the wide diversity of end users. Unlike a hospital IT system used by trained professionals, a wellness app might be used by people aged 8 to 80, with varying levels of tech literacy and physical ability. This calls for inclusive design, meaning ensuring the app is accessible and adaptable to different user needs. For instance, an app for medication reminders should consider that some users might prefer visual schedules, others need auditory alarms, and some might benefit from integration with smart home devices - flexibility can greatly enhance usability.

The integration of mHealth tools into users’ daily lives and broader healthcare systems remains a critical yet often under-addressed dimension of digital health design. For example, if a patient uses a blood pressure tracking app, is there a mechanism for their doctor or nurse to review that data? If not, the patient may feel the effort is not worthwhile. HCD in mHealth is increasingly looking at connecting the dots between personal health tech and formal healthcare. This might involve designing apps that produce outputs easily shareable with clinicians (using understandable charts or generating summary reports at clinic visits), or that allow family members to assist in monitoring, thereby building a support network around the user. A human-centered requirement might be: the solution should not add a burden to clinicians. If an app expects doctors to log into a separate portal to check patient data, it likely won’t be used; a better design would integrate that data into the existing medical record workflow or send alerts only when action is needed. Some recent projects, for example, use a buddy system where a family member gets notified if an elderly patient hasn’t entered data for a while - this was born from user research showing that shared responsibility can improve adherence for some individuals. The human-centered mindset asks: What real-life factors will affect usage of this app, and how can we design for them? Cultural tailoring is another factor where health attitudes differ across communities, so involving users from the target demographic can guide content and design that is culturally sensitive and thus more effective.

A practical illustration of HCD in mHealth can be found in the development of an application aimed at reducing fall risk among older adults. A traditional approach might just create generic exercise videos and reminders. A human-centered approach, as described in a narrative review of current HCD practices, would start by interviewing older adults and caregivers to understand their routines, fears, and motivators [9]. It might find that fear of falling is tied to anxiety when alone, so the app could incorporate a feature to easily notify a family member when the user is about to exercise, providing psychological safety. Usability testing might reveal that some users struggled to navigate menus, leading designers to simplify the interface to just a few big buttons for core functions. The end result could be an app that older adults in testing described as easy and reassuring to use, as opposed to an early prototype that some found confusing. Indeed, a study on a falls detection system noted that actively involving end-users in design led to an app with significantly higher usability scores and acceptance [9]. Moreover, researchers reported that embedding usability testing as a cornerstone of development, rather than a final checkbox, was key to success, echoing the broader industry shift toward UX-driven product cycles [9].

Wearable health devices such as smartwatches, ECG patches, and continuous glucose monitors introduce a unique set of HCD considerations. Wearables must be physically comfortable and aesthetically acceptable since they are worn on the body. User feedback has often driven improvements like adjustable bands for different wrist sizes, longer battery life to reduce charging hassle, and modes that automatically detect activity to reduce user input burden. A scoping review of sensor-based digital health tech found an interesting gap: while many studies focus on the accuracy of sensor data, fewer address user interaction with the sensor/device itself [11]. For example, how easy is it for a patient with arthritis to put on a fitness tracker? Human-centered evaluations in this domain examine factors such as long-term device adherence, assessing whether users continue to wear the device after three months, and user perceptions, including whether they find the data meaningful. The review suggested expanding usability assessments to include practical aspects and recommended involving secondary users, such as caregivers who assist older individuals with wearables. Another important consideration is the potential for data overload, as wearables can produce large volumes of information. However, raw data alone is not helpful. Effective design translates this information into simple and actionable insights for users. For example, rather than merely displaying step counts and heart rates, an app might provide feedback like 'Today you achieved your activity goal, great job!' or 'Your heart rate was above normal for two hours; think about whether you were feeling stressed or physically active.' These interpretations and coaching elements are increasingly built through HCD by asking users what feedback helps them versus what confuses or demotivates them. 

**Figure 4:**
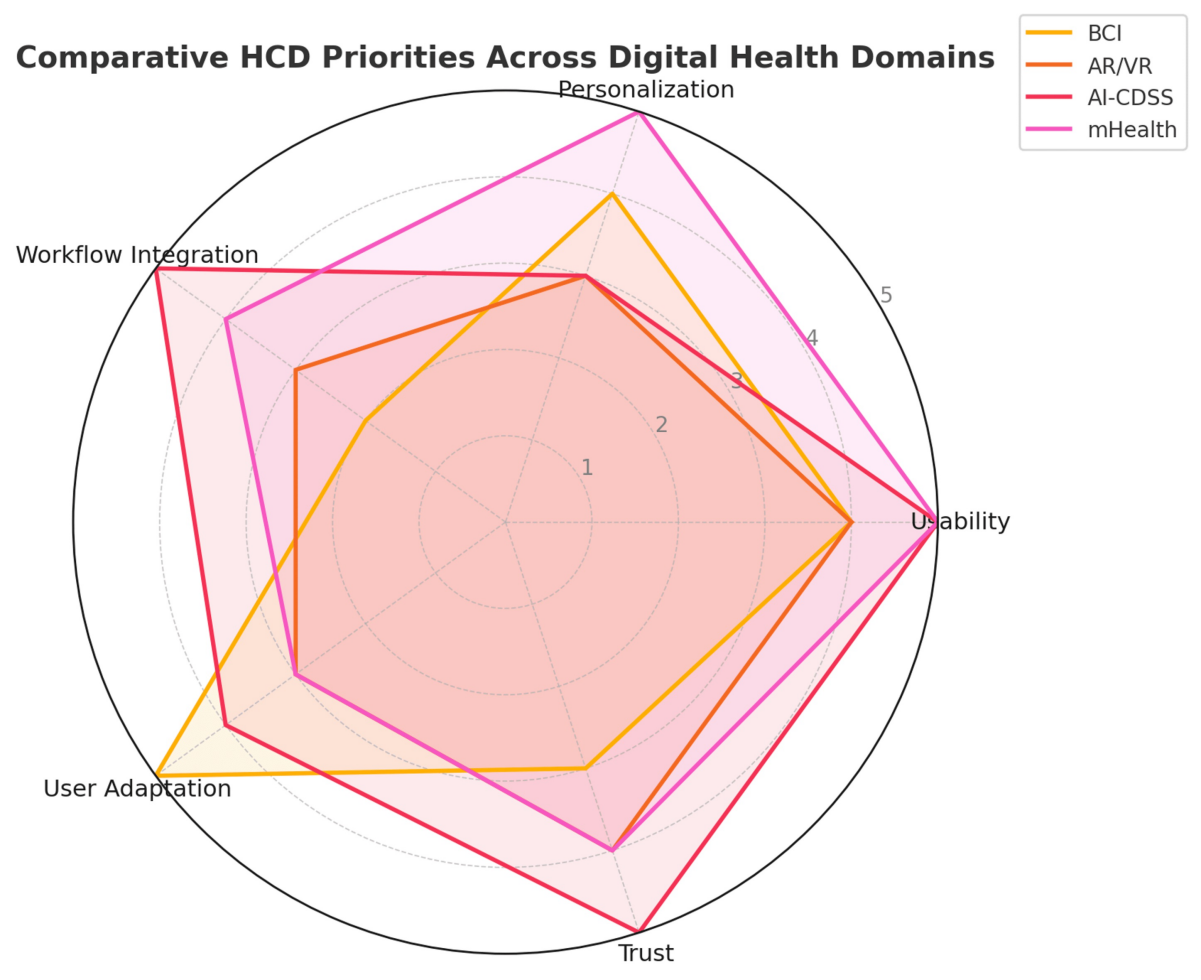
Comparative HCD priorities across digital health domains This radar chart presents a conceptual comparison of key HCD priorities across four major digital health application areas: BCI, AR/VR, AI-CDSS, and mHealth. The five axes — usability, personalization, workflow integration, user adaptation, and trust — represent critical dimensions identified through literature synthesis. Values (scale 1-5) reflect the relative emphasis placed on each factor within each domain, based on thematic analysis of empirical findings and design challenges reported in the reviewed studies. The visualization supports a cross-domain HCD framework for tailoring design strategies to technology-specific needs AI-CDSS: artificial intelligence-based clinical decision support systems; AR/VR: augmented/virtual reality; BCI: brain-computer interface; HCD: human-centered design; mHealth: mobile health Image credits: This is an original image created by the author Katerina D. Tzimourta

These interpretations and coaching elements are increasingly built through HCD by asking users what feedback helps them versus what confuses or demotivates them. As digital health technologies diversify, from mobile apps to wearables and beyond, different domains bring distinct challenges and user needs. To synthesize these variations, Figure 4 presents an illustrative framework that compares HCD priorities across four digital health domains. Each axis represents a core design consideration, scored on a conceptual scale (1-5) reflecting its relative importance or emphasis within each domain. The ratings are not empirical but reflect thematic patterns, implementation challenges, and user-centered demands highlighted in the reviewed sources.

Table 1 presents four core domains in which HCD principles are applied within digital health. For each domain, BCIs, AR/VE, mHealth, and AI-CDSS, key design priorities and challenges are outlined. These include aspects such as usability, personalization, workflow integration, user adaptation, and trust, which must be addressed to ensure effective, ethical, and context-sensitive technological solutions. This typology provides a practical lens to guide HCD strategies tailored to each technological context, while also enabling comparative insights across domains.

**Table 1:** Comparison of HCD considerations across digital health technologies AI-CDSS: artificial intelligence-based clinical decision support systems; AR/VR: augmented/virtual reality; BCI: brain-computer interface; HCD: human-centered design; mHealth: mobile health; XAI: explainable artificial intelligence

Domain	Usability focus	Human-technology interaction	Ethical issues	Emerging trends	Barriers and evidence gaps
BCI	Training, mental effort	Co-adaptive interfaces	Neurodata privacy	BCI + AR integration	High cognitive load, poor generalizability, and limited user studies
AR/VR	Comfort, immersion	Multisensory interfaces	Disorientation	Personalized VR rehab	Cybersickness, lack of long-term user data, and cost/access barriers
AI-CDSS	Workflow fit, trust	Human-AI decision synergy	Explainability	XAI, active learning	Trust issues, integration difficulty, and lack of clinician engagement in design
mHealth	Simplicity, personalization	Daily-life integration	Data sharing, consent	Adaptive feedback, wearables	Low sustained use, poor inclusivity, and insufficient clinical validation

Ethical and legal considerations in human-centered digital health design

Innovations in digital health raise not only technical and user-experience challenges, but also profound ethical and legal questions. A human-centered approach inherently includes an ethical dimension, respecting users’ rights, values, and autonomy. As we design socio-technical health systems, we must ensure that human-centered also means ethically centered, especially when dealing with sensitive personal data, AI-driven decisions, or technologies that interface directly with the human body and mind, like BCIs. 

Data privacy and security are major issues. Digital health tools often collect and process intimate data, such as heart rates, GPS locations, genomic information, and even neural signals. Ensuring privacy and data protection is paramount to maintain user trust and prevent harm. From a regulatory standpoint, frameworks like the EU’s GDPR provide guidelines for data consent, access, and anonymization, but emerging forms of data can fall through the cracks [24]. For instance, brain signals and biometric data collected continuously by wearables present new privacy challenges. A recent article on regulating neural data noted that current laws have not covered neural data clearly and thus may be incapable of fully protecting what some scholars term mental privacy [16]. Neurodata is uniquely sensitive; it can potentially reveal mental states or intentions, leading to concerns about misuse or unauthorized access. Brain-computer interface researchers have explicitly flagged that privacy and security of EEG data, as well as potential misuse by unauthorized parties, are major ethical issues that need to be addressed alongside technical development [25].

HCD principles dictate that users should have transparency and control over their data. This means interfaces that clearly communicate what data is being collected and why, and easy-to-use settings to adjust data sharing preferences. It also means obtaining truly informed consent. However, one challenge identified in the context of big data and AI is static consent: users may give one-time consent for data use, but then the data might be reused in ways they didn’t anticipate. Legal scholars argue for more dynamic consent models or specific provisions for neural data. For example, California and Colorado have pioneered state laws that explicitly categorize neurodata as a protected class, though even these have not provided special additional rules for neural data processing, leaving gaps such as how to handle de-identified brain data or data shared for research [24]. In practice, a human-centered ethic would involve regularly re-engaging users about their data consent, especially if new types of analysis or sharing are considered, and incorporating privacy-by-design techniques like local data processing on the device, end-to-end encryption, and data minimization (collect only what is needed for the feature to work).

User autonomy and informed consent are other significant points. Digital health tools, particularly AI systems or persuasive health apps, can influence user decisions. Ensuring these technologies respect user autonomy is essential. For example, an AI decision support might recommend a treatment - it should ideally explain itself and allow the clinician to disagree freely, rather than subtly coercing a decision. In patient-facing apps, this extends to how nudges and alerts are designed. HCD means designing these nudges to be helpful, not manipulative or overly intrusive. For instance, a medication app that nags a user in a shaming tone could be considered ethically problematic and counterproductive; a more respectful design might encourage with positive reinforcement and allow the user to set their own reminder schedule. Informed consent in digital health is an ongoing concern. Many apps and devices have lengthy privacy policies or terms that users rarely read. Simplifying consent materials and using just-in-time notices (e.g., a prompt that appears when the app is about to use location data, explaining the purpose) can help users make informed choices. From a legal perspective, regulators are looking at how to update consent paradigms for AI, where even the developers may not fully know how data will be processed in the future as models evolve. One emerging concept is the right to mental integrity and privacy, which international bodies like the OECD and UNESCO have discussed in the context of neurotechnology [21]. While these are not laws yet, these ideas suggest that designers and engineers should treat neural and biometric data with special care, possibly giving users opt-in choices for each distinct use of such data.

Furthermore, bias, fairness, and inclusion should be taken into account. AI systems in health have faced scrutiny for biases; for example, an algorithm was found to underestimate the severity of illness in patients of color because it used health expenditure as a proxy for health needs. Ethical HCD requires proactively addressing such biases. This can mean curating diverse and representative data for model training, and also allowing users to detect and report potential biases. For instance, if a clinical AI consistently recommends a less aggressive treatment for women compared to men, clinicians using it should have avenues to flag this discrepancy. Incorporating fairness checks into the design and validation of algorithms is now recommended practice [6]. From an HCD standpoint, diversity in the design team and test user group is important to catch biases - having clinicians of different backgrounds, and patient advocates, involved in evaluating an AI can surface concerns that a homogeneous team might miss. Fairness also ties into accessibility: an ethical digital health product should not unduly exclude certain groups. Consider telehealth platforms - if they are not designed with multilingual support or compatibility with screen readers for the visually impaired, they risk exacerbating health disparities. Legal frameworks like the Americans with Disabilities Act (ADA) increasingly are interpreted to cover digital tools, meaning a lack of accessibility could be a legal violation. Thus, inclusive design (a pillar of HCD) is not just a nice-to-have but part of ethical and legal responsibility.

On the other hand, responsible innovation and stakeholder engagement are also important. The concept of responsible research and innovation (RRI) in health echoes many HCD ideals. It emphasizes aligning innovation with societal values and ethics by involving stakeholders from an early stage [26]. A policy-oriented framework for responsible innovation in health identifies dimensions like population health impact, equity, and sustainability as key evaluative criteria [26]. In practical terms, this means when designing a new digital health solution, one should ask: Will this improve outcomes for those who need it most? Could it inadvertently worsen disparities? Is it sustainable and scalable in the healthcare system? For example, a fancy AI app might help tech-savvy urban patients but be unusable for patients in rural or low-resource settings; responsible innovation would flag that as an issue to address. Involving a broad range of stakeholders, such as patients, clinicians, and policymakers, can ensure that broader impacts are considered and integrated into the design requirements. Lehoux et al. argue that responsible innovation provides a common framework to ensure health technologies tackle real system-level challenges like accessibility and cost, not just niche tech problems [26]. An example of this principle in action is the development of contact tracing apps during COVID-19: some countries adopted a very centralized surveillance approach that raised privacy concerns, whereas others (often following more public engagement and expert consultation) chose decentralized, privacy-preserving designs. The latter had more public trust and uptake, demonstrating that building ethical principles into design can directly impact adoption [27].

Also, regulatory compliance and design are significant issues [28]. Navigating the regulatory landscape is a practical aspect of ethical design. Medical device regulations (like FDA or CE marking processes) increasingly cover software and algorithms. Human-centered evidence, such as usability test results and human factors engineering reports, is often required in submissions to demonstrate safety. For instance, the FDA expects usability testing for devices to ensure that use errors (mistakes in using the interface that could harm patients) have been mitigated through design [29]. This is where HCD and regulation align: doing thorough user testing and iterating reduces the risk of dangerous errors, which not only is ethically right but also satisfies regulatory scrutiny. Data protection regulations necessitate the inclusion of functionalities such as data export and deletion, which must be integrated into system design from the early stages. Users should have the ability to access, transfer, and permanently remove their personal data, while also being informed about who has accessed their information through transparency mechanisms such as access logs or reports. Although these features may be deprioritized in typical development cycles, they are fundamental for ensuring user trust and regulatory compliance. A survey of digital health consumers might show, for example, that a significant portion worry about what happens to their data; addressing that through clear privacy settings and information can be a market differentiator as well as an ethical imperative [30].

In summary, the ethical and legal context forms an important backdrop for HCD in digital health. Privacy, consent, fairness, and accountability are not abstract principles; they manifest in concrete design decisions on how we ask for permission, how we explain AI decisions, how we secure data, and how we include all users. The human-centered mindset naturally extends to these domains: just as we empathize with users to improve usability, we should empathize with their rights and concerns to uphold ethics. By doing so, we not only create technologies that people can use, but also technologies people can trust. In the long run, integrating ethical deliberation and stakeholder engagement into the design process leads to more sustainable innovations that policy-makers and the public will support. Responsible, human-centered innovation is thus about designing the future of digital health with care: care for the user, care for the societal impact, and care for the values that define quality healthcare.

Limitations

As a narrative review, this work does not adhere to a formal systematic review protocol. Its scope and thematic coverage are guided by conceptual relevance rather than predefined inclusion criteria, which may limit reproducibility and completeness. Despite efforts to capture a broad cross-section of domains and perspectives, certain digital health areas or regional implementations may not be fully represented. Moreover, a meta-analysis was not conducted due to the methodological and topical heterogeneity of the included sources, which span design studies, usability evaluations, and conceptual discussions. Additionally, while ethical and regulatory considerations were integrated throughout the review, the rapidly evolving nature of these topics may result in partial coverage. Future studies could build upon this synthesis by incorporating empirical mapping or stakeholder consultation to expand the scope and ensure broader applicability.

## Conclusions

HCD in socio-technical systems for digital health is not a one-time task or a single methodology - it is a comprehensive ethos that must permeate the entire lifecycle of technology development and deployment. This review explored how HCD, informed by systems theory, ergonomics, and information systems research, provides both a conceptual framework and a practical toolkit to tackle the complexity of digital health innovations. Across diverse applications - BCIs that translate thoughts to action, AR/VR systems that create new clinical realities, AI decision aids that analyze vast data, and mHealth apps that accompany us in daily life - we found a common theme: technology succeeds when it serves human users in context, addressing their real needs, capabilities, and limitations.

Each domain presented unique challenges but also illustrated how HCD approaches can lead to better outcomes. As digital health continues to evolve with advances like smarter AI, more immersive XR, and high-bandwidth brain interfaces, the role of human-centered, socio-technical design will only become more vital. We hope that the insights summarized in this review encourage researchers, designers, and healthcare leaders to adopt and champion HCD approaches. By doing so, we can ensure that the next generation of digital health tools truly center on the humans they intend to serve, leading to safer, more effective, and more humane healthcare in the digital age. Of note, this review goes beyond core HCD principles by synthesizing their application in emerging contexts, such as the integration of AR with BCI control, the ethical handling of neurodata, and the design of explainable AI systems in clinical workflows. These cross-domain insights aim to support a more nuanced and future-facing understanding of HCD in digital health.
